# Altered Glycogen Metabolism in Cultured Astrocytes from Mice with Chronic Glutathione Deficit; Relevance for Neuroenergetics in Schizophrenia

**DOI:** 10.1371/journal.pone.0022875

**Published:** 2011-07-28

**Authors:** Suzie Lavoie, Igor Allaman, Jean-Marie Petit, Kim Q. Do, Pierre J. Magistretti

**Affiliations:** 1 Department of Psychiatry, Centre for Psychiatric Neuroscience, University Hospital Centre and University of Lausanne, Lausanne, Switzerland; 2 Laboratory of Neuroenergetics and Cellular Dynamics, Brain Mind Institute, Ecole Polytechnique Fédérale de Lausanne (EPFL), Lausanne, Switzerland; Rikagaku Kenkyūsho Brain Science Institute, Japan

## Abstract

Neurodegenerative and psychiatric disorders including Alzheimer's, Parkinson's or Huntington's diseases and schizophrenia have been associated with a deficit in glutathione (GSH). In particular, a polymorphism in the gene of glutamate cysteine ligase modulatory subunit (GCLM) is associated with schizophrenia. GSH is the most important intracellular antioxidant and is necessary for the removal of reactive by-products generated by the utilization of glucose for energy supply. Furthermore, glucose metabolism through the pentose phosphate pathway is a major source of NADPH, the cofactor necessary for the regeneration of reduced glutathione. This study aims at investigating glucose metabolism in cultured astrocytes from GCLM knockout mice, which show decreased GSH levels. No difference in the basal metabolism of glucose was observed between wild-type and knockout cells. In contrast, glycogen levels were lower and its turnover was higher in knockout astrocytes. These changes were accompanied by a decrease in the expression of the genes involved in its synthesis and degradation, including the protein targeting to glycogen. During an oxidative challenge induced by *tert*-Butylhydroperoxide, wild-type cells increased their glycogen mobilization and glucose uptake. However, knockout astrocytes were unable to mobilize glycogen following the same stress and they could increase their glucose utilization only following a major oxidative insult. Altogether, these results show that glucose metabolism and glycogen utilization are dysregulated in astrocytes showing a chronic deficit in GSH, suggesting that alterations of a fundamental aspect of brain energy metabolism is caused by GSH deficit and may therefore be relevant to metabolic dysfunctions observed in schizophrenia.

## Introduction

Cellular respiration is an essential process during which the energy derived from the oxidation of carbon atoms contained in a molecule of glucose or its metabolites are used to produce ATP. During this vital process, which involves the electron transport chain in mitochondria, reactive oxygen species (ROS) are normally produced [1]. These molecules are highly reactive and can cause cell membrane [2] and DNA damage [3], leading to cell death if they are not adequately removed.

The brain consumes 20% of total body oxygen and utilizes 25% of total body glucose, and hence this organ produces large amounts of ROS [For a review on brain metabolism see 4]. Consequently, antioxidant defenses are of primary importance to avoid oxidative stress and thus maintain normal brain functions. Among the endogenous antioxidant defense mechanisms the GSH system is the major cellular antioxidant and redox regulator in the brain and its presence is fundamental for the protection against oxidative stress induced by ROS and other oxidising molecules [5]–[8].

The GSH redox system and glucose metabolism are clearly linked, with GSH being necessary for the removal of the by-products of glucose utilization for energy production. Furthermore, the pentose phosphate pathway (PPP), a pathway through which glucose can be metabolized, is a major source of NADPH, a cofactor necessary for the recycling of the oxidised GSH (GSSG) [7]. During oxidative stress, the restoration of the initial high ratio of GSH to GSSG depends on the synthesis of NADPH by glucose-6-phosphate dehydrogenase (G6PD) [9]–[11], the first and rate-limiting enzyme of the PPP, and on the presence of glucose [12],[13]. Indeed, if glucose levels are decreased or if G6PD is inhibited, there is a subsequent reduction in NAPDH levels leading to a decrease in the GSH/GSSG ratio, ultimately resulting in oxidative stress and potentially in apoptosis [14]. However, to our knowledge, the opposite effect, i.e. the impact of a deficit in GSH on glucose utilization, has never been reported. Considering that glucose metabolism leads to the production of ROS, we hypothesize that an organism presenting with chronically low GSH levels is likely to show adaptations regarding its energy use. GSH deficits have been observed in several neurodegenerative and psychiatric disorders including Alzheimer's, Parkinson's or Huntington's diseases [For reviews see 15], [16] as well as in schizophrenia [17]–[19]. In particular, a polymorphism in the gene of the modulatory subunit of glutamate cysteine ligase (GCL), the limiting enzyme in the synthesis of GSH, is associated with schizophrenia [20]. Understanding the consequences of such a deficit in these clinical conditions is essential to better focus potential therapies.

Astrocytes are fundamental to both antioxidant defense and energy metabolism in the brain [21]. Indeed, GSH levels are higher in astrocytes than in neurons and increasing GSH in astrocytes protects neurons against oxidative stress [22], [23]. Astrocytes also represent the prevalent site of glucose entry into the brain and they have the capacity to sense synaptic activity and dynamically couple it to substrate distribution to neurons [24]–[26]. Indeed, extracellular lactate released by astrocytes following glucose metabolism can be subsequently used by neurons to meet their energy needs [27], [28]. Furthermore, glycogen, which represents the major form of glucose storage, is almost exclusively found in astrocytes in the adult brain. Glycogen metabolism is under the control of a restricted number of neurotransmitters and modulators [29]–[35]. Experiments indicate that glycogen mobilization can support neuronal viability in case of energy depletion [36], [37], and it is also implicated in normal brain function. For instance, astrocytic glycogenolysis is increased during early memory consolidation in the chicken brain [38], [39], action potential propagation in the mouse optic nerve to support the energy needs of axons [40], [41] and following sensory stimulation [42]. It has also been demonstrated that the presence of glycogen is essential for proper glutamatergic neurotransmission, i.e. vesicular release and subsequent uptake [43]. Recent results indicate that glycogen mobilization and the associated increase in lactate release by astrocytes is required for the establishment of long-term memory [44].

GSH is synthesized following two reactions catalysed by GCL and GSH synthetase (GSS). GCL is a heterodimer made of a catalytic subunit (GCLC), which contains the substrate's binding sites, and a modulatory subunit (GCLM). The latter modulates the affinity of GCLC for substrates and inhibitors [45]–[47]. The *gclm* knockout (KO) mouse represents a good model to study a chronic GSH deficit [48], [49], since it shows decrease in GSH levels of at least 80% in liver, lung, pancreas and blood [50], as well as in astrocytes [51]. Given the interplay between the glucose and GSH metabolic pathways, the aim of the present study was to investigate glucose metabolism and the response to oxidative stress in cultured astrocytes from the GCLM-KO and wild-type (WT) mice. Our results show that glycogen status and utilization are clearly modified in astrocytes from GCLM-KO mice, and these observations could be relevant to neuroenergetics impairments in schizophrenia.

## Materials and Methods

### Ethics Statement

All experiments were performed in accordance with the guidelines outlined in the *Guide for the Care and Use of Laboratory Animals* (Swiss National Research Council). Approval #2091 was given on March 13^th^ 2008 by the local Veterinary Office (Service de la Consommation et des Affaires Veterinaires, Vaud canton, Switzerland) for studying the effects of a deficit in glutathione in cultured astrocytes from GCLM-KO mice and their corresponding WT.

### Materials

GCLM-KO mice, backcrossed with C57BL/6J mice over more than 10 generations, were kindly provided by Timothy P. Dalton and Ying Chen (Center for Environmental Genetics, Cincinnati, OH, USA) [50], and were bred in our animal facility.

Unless otherwise stated, all chemicals were provided by Sigma-Aldrich (St-Louis, MO, USA).

### Primary cultures of cortical astrocytes

Astrocytes cultures from P1-2 C57BL/6 WT and GCLM-KO mice were prepared as previously described [52], [53]. Cortices were dissected in DMEM medium (Invitrogen, Carlsbad, CA, USA) containing 25 mM glucose and supplemented with 10% foetal calf serum (BioConcept, Allschwil, Switzerland) and penicillin (100 u/ml)/streptomycin (100 µg/ml). Cortical cells were mechanically dissociated through needles with decreasing diameters and resuspended in the supplemented DMEM medium. Astrocytes were plated on 35-mm poly-L-ornithine-coated dishes and left to grow for two weeks at 37°C in a humidified 5% CO_2_ atmosphere. Under these conditions, more than 95% of the cells were immunoreactive to glial fibrillary acidic protein (GFAP, astroglial marker) [54]. Twice a week old medium was replace by 2.5 ml of fresh medium. Under these conditions, the purity of the astrocytes cultures is higher than 95% [54].

### Experimental design

Twenty-four hours before any treatment or measurement, the culture medium was removed and astrocytes were incubated in 2 ml of glucose-free DMEM supplemented with 5 mM glucose, 44 mM NaHCO3, 4 mM L-glutamine and 10 ml/l of penicillin-streptomycin solution (DMEM5). In a first set of experiments, the baseline metabolic status of WT and KO astrocytes was assessed by measuring the rate of 2-deoxy-D-glucose (2DG) uptake and glycogen levels. These measurements were also done in the presence of 1,4-dideoxy-1,4-imino-d-arabinitol (DAB), an inhibitor of glycogen phosphorylase [55] that was added to the medium for 1 hour. Lactate released from the cells and CO_2_ produced through the PPP and the tricarboxylic acid (TCA) cycles were also measured. In a second series of experiments, oxidative stress was induced in both WT and KO astrocytes by adding *tert*-Butylhydroperoxide (tBH; 10 µM and 50 µM) to the medium for 40 minutes. tBH is a ROS-generating agent that was shown to rapidly engage the GSH redox system [56]. Following this short oxidative challenge, GSH and GSSG levels, 2DG uptake and glycogen mobilization were measured. The metabolic response of cells to the oxidative stress was also measured in the presence of DAB, which was added to the medium 1 hour before and maintained during tBH treatment. At the end of every treatment, cell death was evaluated by measuring the activity of lactate dehydrogenase (LDH) released into the medium by dying cells.

### Glycogen assay

After appropriate treatment, cells were washed three times with ice-cold PBS and sonicated in 30 mM HCl. The suspension was used to measure glycogen as previously described [53]. In a first 100-µl aliquot, 300 µl of sodium-acetate buffer (0.1 M, pH 4.6) was added. In a second aliquot, 300 µl of the same buffer containing 1% (v/v) of amyloglucosidase (10 mg/ml; Roche Diagnostics, Rothkreuz, Switzerland) was added. Aliquots were incubated at room temperature (RT) for 30 min. Then, 2 ml of Tris-HCl buffer (0.1 M; pH 8.1; 3.3 mM MgCl_2_, 0.2 mM ATP, 30 µM NADP, containing 0.7 U/ml of hexokinase and 0.35 U/ml of glucose 6-phosphate dehydrogenase (HK-G6PD; Roche Diagnostics) were added, and the mixture was incubated at RT for 30 min. Fluorescence associated with the NADPH formed was then read on a fluorometer (excitation: 340 nm; emission: 450 nm) after calibration with an appropriate standard curve using glucose as standard. The first aliquot gives the sum of glucose and glucose 6-phosphate, and the second gives the sum of glycogen, glucose and glucose-6-phosphate; the amount of glycogen is determined by the subtracting the two results. A third aliquot (20 µl) was used to measure the protein content using the Advance Protein Assay (APA; Cytoskeleton, Denver, CO, USA). Results are presented in nmol glycogen per mg of protein, one mole of glycogen corresponding to one mole of glycosyl units originating from glycogen.

### 2DG uptake assay

Glucose utilization was determined as previously described [53]. 20 min before the end of the treatments, the medium was replaced by 2 ml of DMEM5 medium containing 2-[1,2^3^H] deoxy-D-glucose ([^3^H]2DG; 30–60 Ci/mmol; Anawa, Wangen, Switzerland) at a concentration of 1 µCi/ml. Substances were maintained in the medium during this period. To stop the reaction the medium was removed (and collected on ice for lactate release assay, see below), cells were rinsed three times with ice-cold PBS, and 2 ml of 10 mM NaOH/0.1% Triton X-100 was added to lyse the cells. 500-µl aliquots were assayed for radioactivity by liquid scintillation counting (Ecoscint XR, National Diagnostics, Atlanta, GA, USA), and a 20-µl aliquot was used for measurement of protein content using the APA. Results, which represent glucose transporters-mediated uptake and subsequent phosphorylation, were calculated by subtracting from total counts the portion that was not inhibited by the glucose transporters inhibitor cytochalasin B (25 µM). [^3^H]2DG uptake, therefore glucose utilization, is expressed in fmol per mg of protein.

### Lactate release assay

Lactate release into the medium was measured enzymatically as previously described [53]. To a 200-µl aliquot of the culture medium (collected during the [^3^H]2DG assay, see above), 1 ml of a glycine-semicarbazide buffer (0.2 M; pH 10; NAD 3 mM, LDH 14 U/ml; Roche Diagnostics) was added. Samples were incubated at 40°C for 1 h. After samples cooled down to RT, fluorescence associated with the NADH formed was read on a fluorometer (excitation: 340 nm; emission: 450 nm), after calibrating the apparatus with a standard curve using sodium L-lactate as standard. Lactate released into the medium is expressed in nmol per mg of protein.

### ^14^CO_2_ production assay

Production of ^14^CO_2_ from D-[1-^14^C]-glucose and D-[6-^14^C]-glucose (specific activities, 50–62 mCi/mmol; GE Healthcare, Otelfingen, Switzerland) was used to determine glucose utilization via the PPP and the TCA cycle, according to published procedure [53]. Culture medium was removed and cells were incubated for two hours in serum-free DMEM containing 2.5 mM glucose, 7.5 mM NaHCO_3_, 5 mM HEPES and penicillin-streptomycin 10 ml/l solution at 37°C in an atmosphere containing 5% CO_2_. At the end of this incubation period, the medium was replaced by 2 ml of the same medium containing 2.3 µCi/ml D-[1-^14^C]-glucose, or D-[6-^14^C]-glucose and culture dishes were placed in sealed glass containers and incubated for 2 hours at 37°C. The reaction was stopped by addition of 500 µl of 0.2 M HCl to the cells and 1 ml Carbo-Sorb (PerkinElmer; Schwerzenbach, Switzerland) on the bottom of the container. After a 1-hour equilibration, containers were opened and two 400-µl aliquots of Carbo-Sorb were assayed for radioactivity by liquid scintillation counting. Production of ^14^CO_2_ from D-[1-^14^C]-glucose reflects the total CO_2_ produced in both PPP and TCA whereas ^14^CO_2_ produced from D-[6-^14^C]-glucose reflects CO_2_ production in the TCA only. CO_2_ production in the PPP is obtained by subtraction. Total CO_2_ production from glucose can be calculated from the molar ratio between unlabelled and labelled glucose. Cell protein content was determined using the BCA protein assay reagent kit (Pierce, Rockford, IL, USA) in parallel on cells from the same culture and results are expressed as nmol of CO_2_ produced per minute per mg of protein.

### Gene expression

Astrocytes were harvested with trypsin-EDTA. RNA was extracted with the Perfect Pure RNA cultured Cell Kit following manufacturer's instructions (5 Prime GmbH, Hamburg, Germany). The concentration of each sample was measured with a NanoDrop (NanoDrop Technologies, Wilington, DE, USA), and the purity was assessed using the ratios of optic density at 260/280 nm and 260/230 nm. The first strand of cDNA was synthesized from 200 ng of total RNA using TaqMan ® RT-reagents (Applied Biosystem, Foster City, USA) after incubation during 45 min at 48°C, followed by 5 min at 95°C and finally stored at 4°C.

Three glycogen-related genes were amplified, i.e. the protein targeting to glycogen (PTG), muscle glycogen synthase (GS) and brain glycogen phosphorylase (GPhos). Cyclophylin was used as a normalization gene. It is worth noting that the muscle GS is used because it is the most common form of the enzyme found in the brain [57]. Primer sequences (Table 1) were designed using primer Express 3.0 software (Applied Biosystems) and oligonucleotides were synthesized by Microsynth (Balgach, Switzerland). Quantitative PCR was performed in an ABI Prism 7900 (Applied Biosystems). The PCR mix was composed of 1.75 µl of RT-products, 1.05 µl of forward and reverse primers (100 µM) and 17 µl of Sybr-Green PCR MasterMix (Applied Biosystem). Data were computed using the sequence detector software SDS 2.3 (Applied Biosystems). To determine the level of expression of the different genes, a macro (ExcelTM software) produced by the genomic platform of the Geneva University (Frontiers in Genetics, UNIGE, Geneva, Switzerland) was used. Results are expressed as the expression levels of a given gene relative to the expression levels of cyclophylin.

**Table 1:** Nomenclature name, GeneBank Accession number and sequences of genes used in the quantification analyses.

Genes	GeneBan	
(nomenclature name)	Accession	Sequences
	Number	
Cyclophyline	NM 008907	Forward: 5′-CAAATGCTGGACCAAACACAA-
(Ppia)		Reverse: 3′-TCTGACTTACCGACCTACCG-
PTG	NM 016854	Forward: 5′-TGCCTCTCGGTCCAATGAG-
(Ppp1r3c)		Reverse: 3′-AACTGTTCAAGGCAGTACGG-
GSynt	NM 030678	Forward: 5′ GCTGGACAAGGAGGACTTCACT-
(Gys1)		Reverse: 3′-CAGAAAGGGTGGTCACACGT-
GPhos	NM 153781	Forward: 5′-GCTGCTCAACTGCCTACACATT-
(Pygb)		Reverse: 3′-GGAAACACGGGTCCTGACAA-

### Determination of oxidized and reduced GSH

Quantification of total GSH and oxidized GSH (GSSG) was performed with an assay based on the Tietze method [58] and modified as described [59]. Cells were washed, scraped out of the dish and sonicated before an aliquot was reserved for subsequent analysis of protein content using the APA. Proteins in the remaining cell solution were precipitated with 5-sulfosalicylic acid and removed by centrifugation. The pH of the supernatant was adjusted with triethanolamine. For GSSG measurement, an aliquot of the supernatant was incubated for 45 to 60 min at room temperature with 2-vinylpyridine, which forms a stable complex with reduced GSH [60]. Sample aliquots were mixed with a buffer containing 0.25 U/ml GSH reductase from baker's yeast, 0.25 mM EDTA, 0.075 mM 5,5′-dithiobis(2-nitro-benzoic acid) (DTNB) and 0.075 mM NADPH. The rate of increase in absorbance at 405 nm, which measures the reduction of DNTB by GSH, was proportional to the total GSH or to the amount of GSSG when 2-VP was added. Total GSH content in cells are expressed in nmol GSH/mg protein. GSSG levels are presented as a percentage relative to reduced GSH (reduced GSH levels = total GSH levels - 2×GSSG levels).

### Assessment of cell viability

Cell viability was assessed by measuring the activity of LDH released in the culture medium by dying cells [Adapted from 61]. The decrease in NADH in the presence of pyruvate is proportional to the LDH activity. This can be quantified by measuring the absorbance at 340 nm. The amount of LDH in the medium after each treatment was reported to the total amount of LDH released from cells after a freeze-thaw cycle. Results are presented as the percentage of cell death relative to total cell death.

### Statistical analyses

Statistical analyses were performed using the SPSS software. Normal distribution of data was verified with the Shapiro-Wilk test. Comparisons between WT and KO were done using the t-test for independent samples when the normal distribution of data and equality of variance criteria were fulfilled; otherwise, the non-parametric Mann-Whitney test was used. The effect of treatments was assessed with a one-way ANOVA with Treatment as group factor, or a two-way ANOVA with DAB and Treatment as between factors, followed by the Bonferroni's *post hoc* multiple comparison. For all statistical tests, significant probability level was set to p≤0.05 and data were presented as the mean ± SEM.

## Results

### Characterization of glucose and glycogen metabolism in WT and GCLM-KO astrocytes

**Figure 1:**
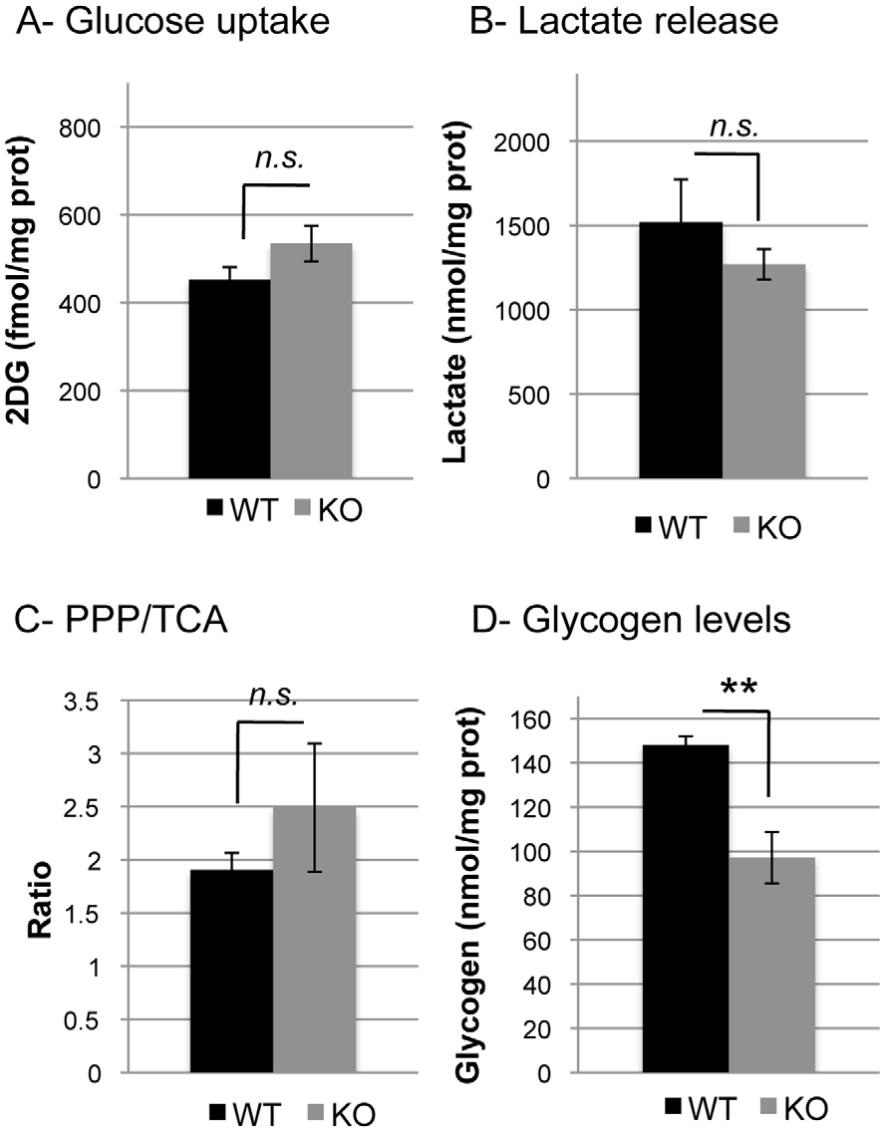
Baseline characterization of WT and GCLM-KO astrocytes. A- there was no significant difference between WT and KO astrocytes in glucose utilization as assessed by measuring the [^3^H]2DG uptake rate during 20 min; n = 6 in WT and n = 11 in KO. B- no significant difference was observed between WT and KO astrocytes in the amount of lactate released from the cells into the medium during 20 min; n = 9 in WT and n = 8 in KO. C- there was no significant difference between WT and KO astrocytes in their preference for glucose to go through the PPP or the TCA. This was assessed by measuring the CO_2_ released from the two pathways over a 2-h period, and results are presented as the amount of CO_2_ released from the PPP relative to the amount coming from the TCA. The mean of individual ratio is presented. n = 6. D- glycogen levels were lower in KO compared to WT astrocytes; n = 9 in WT and n = 8 in KO. ** p<0.01; *n.s.*, non significant.

Figures 1A and B show that there was no difference in the rate of glucose utilization, as assessed by the [^3^H]2DG uptake, and the release of lactate into the medium between WT and KO astrocytes. In order to reveal any changes in glucose metabolism through the PPP or TCA cycles, CO_2_ production by both pathways was determined. Fig. 1C shows that there was no difference between WT and KO cells in the PPP/TCA ratios. In addition, when looking at the amount of CO_2_ formed via each of these pathways, results show that there is no significant difference between WT and KO astrocytes. Indeed, CO_2_ production through the PPP is 0.073±0.006 nmol/mg prot/min in WT and 0.081±0.013 nmol/mg prot/min in KO, and CO_2_ production through the TCA cycle is 0.039±0.003 nmol/mg prot/min in WT and 0.038±0.005 nmol/mg prot/min in KO. On the other hand, glycogen levels were significantly lower in KO cells (−34.4%; p = 0.009; Fig. 1D).

**Figure 2:**
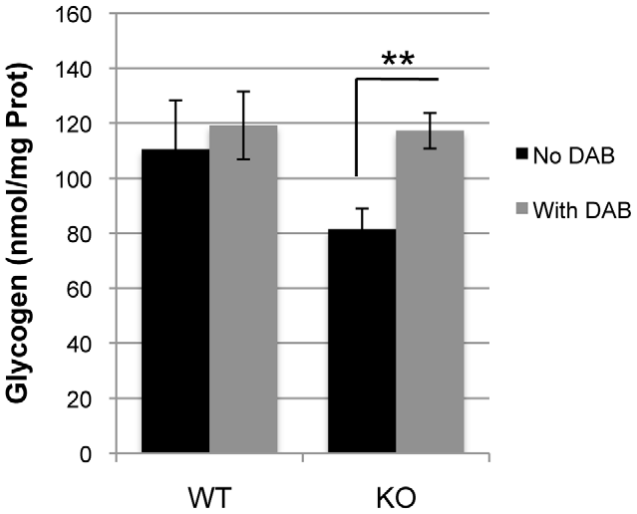
Effect of DAB on glycogen levels in WT and GCLM-KO astrocytes. In the presence of 500 µM DAB for 24 hours, glycogen levels were increased in KO astrocytes, but not in WT. n = 9, except for KO with DAB where n = 7. ** p<0.01.

**Figure 3:**
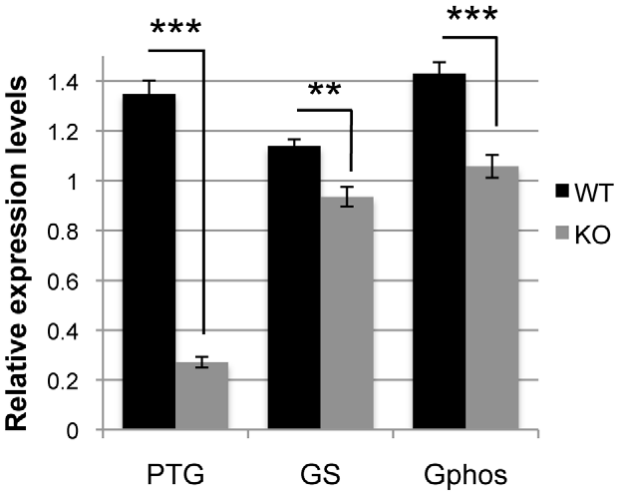
Basal expression of glycogen-related genes in WT and GCLM-KO astrocytes. Expression was lower in KO compared to WT astrocytes for all three genes studied. n = 9. ** p<0.01; *** p<0.001.

Low glycogen levels, as observed in KO (Fig. 1D), could be indicative of a change in turnover. To test this hypothesis, a 24-h treatment with 500 µM DAB, a selective inhibitor of GPhos, was applied. Glycogen levels were significantly increased in KO (1.4-fold increase; p = 0.004), while no change was observed in WT (Fig. 2). These results demonstrate that, at baseline, glycogen turnover seems higher in the KO astrocytes than in WT cells. We then aimed to determine if such an increase in glycogen turnover was accompanied by modifications in the expression of genes involved in astrocyte glycogen metabolism [62], [63]. Figure 3 shows that the relative gene expression levels of PTG, GS and GPhos are lower in KO as compared to WT (−79.9%, p<0.001; −26.1%, p<0.001; −18.3%, p = 0.003, respectively). Therefore, in spite of lower amounts of these synthesizing and phosphorylating enzymes, a higher glycogen turnover appears to be observed in KO suggesting an increase in the activity of these enzymes.

### Induction of an oxidative stress

**Figure 4:**
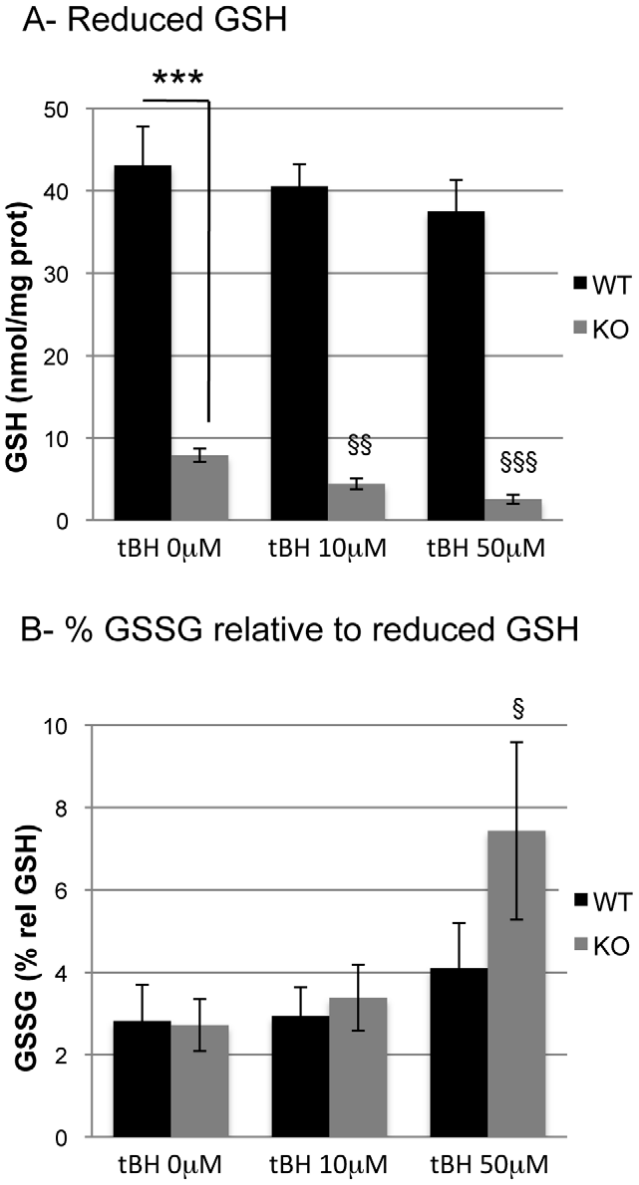
Effect of tBH treatment in GSH and relative GSSG levels in WT and GCLM-KO astrocytes. A- reduced GSH levels were highly significantly lower in KO than in WT astrocytes and tBH treatment led to a further decrease in GSH levels in KO astrocytes without significantly affecting these levels in WT. B- percentage of GSSG relative to reduced GSH were significantly increased compared to control only after treatments with tBH 50 µM in GCLM-KO astrocytes. n = 9. § p<0.05 vs no tBH; §§ p<0.01 vs no tBH; §§§ p<0.001 vs no tBH; *** p<0.001 vs other genotype.

More subtle differences between WT and GCLM-KO can be revealed following oxidative stress [64]. Astrocytes were treated with low concentrations of tBH (10 µM and 50 µM) for 40 minutes to induce an oxidative challenge without increasing cell death. As previously observed [51], prior to treatment, the GSH levels were much lower in the KO compared to the WT (−81.6%; p<0.001; Fig. 4A), while the percentage of GSSG relative to GSH was not significantly different (Fig. 4B). After 40-min treatments with 10 µM and 50 µM tBH, there was no significant effect on both GSH and GSSG levels in WT astrocytes (Fig. 4A and Fig. 4B). In contrast, treatment with either 10 µM or 50 µM tBH led to a decrease in GSH levels in the KO cells (−44.1%, p = 0.004 and −67.9%, p<0.001, respectively; Fig. 4A). Furthermore, treating KO astrocytes with 50 µM tBH induced an increase the amount of GSSG relative to GSH (3.5-fold increase; p = 0.03; Fig. 4B).

It is important to note that assessment of cell death by measuring LDH release in the medium showed no difference between WT and KO (data not shown). Furthermore, a two-way ANOVA showed no significant increase in LDH release after treatments with DAB, 10 µM tBH and 50 µM tBH (data not shown).

### Differential use of glycogen and glucose following oxidative stress

**Figure 5:**
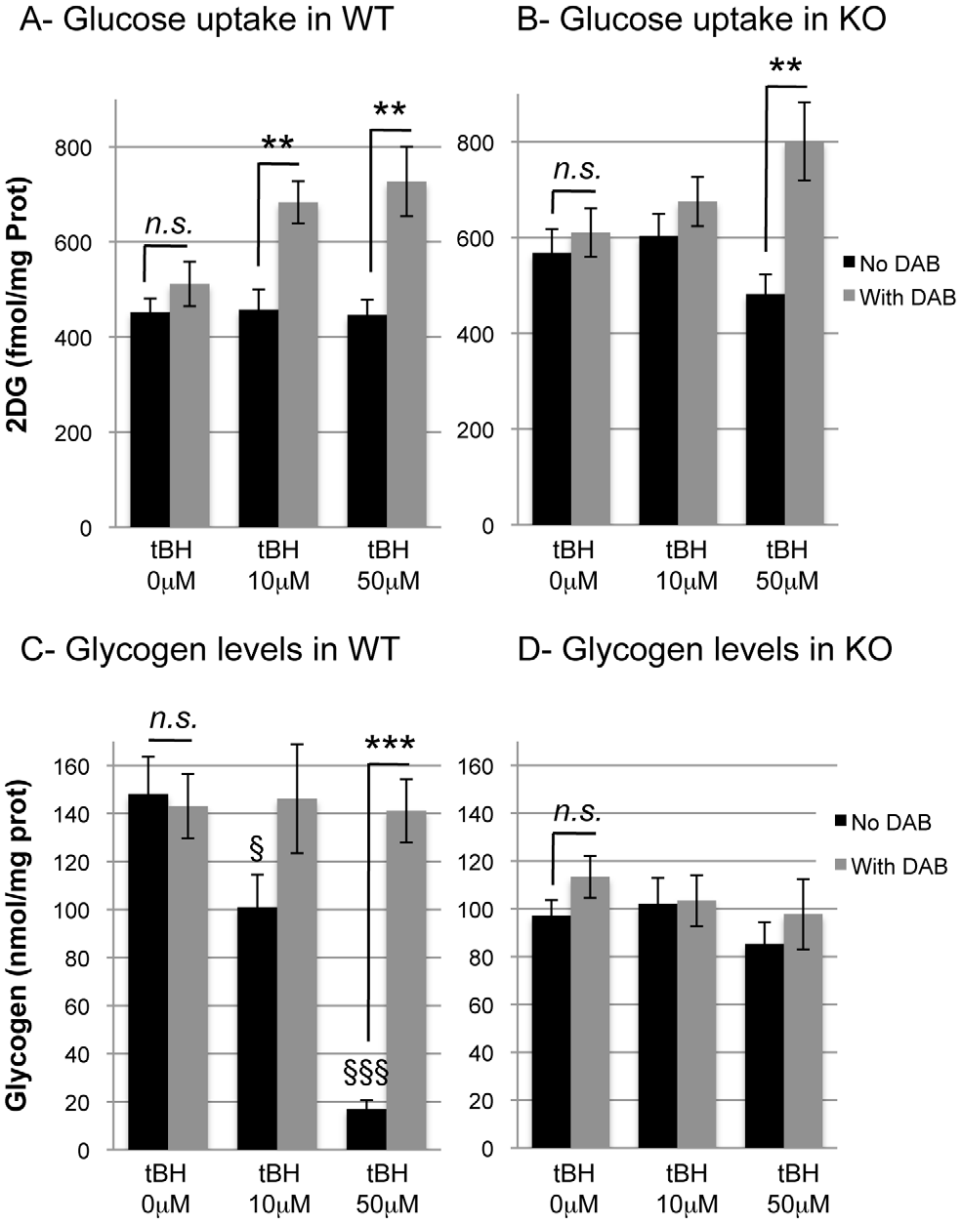
Effect tBH and DAB on glucose utilization and glycogen mobilization in WT and GCLM-KO astrocytes. A- glucose utilization was increase in WT astrocytes following a 40-min treatment with 10 µM and 50 µM tBH in the presence of DAB. n = 6. B- glucose utilization was increase in KO astrocytes following a 40-min treatment with 50 µM tBH in the presence of DAB. n = 11–12. C- glycogen levels were not changed following DAB treatment, but were highly significantly decreased following 40-min tBH treatment in WT astrocytes. n = 9. D- glycogen levels were not significantly affected by either DAB or tBH treatment in GCLM-KO astrocytes. n = 6–9. § p<0.05 vs no tBH; §§§ p<0.001 vs no tBH; ** p<0.01 vs other genotype; *** p<0.001 vs other genotype; *n.s.*, non significant.

**Figure 6:**
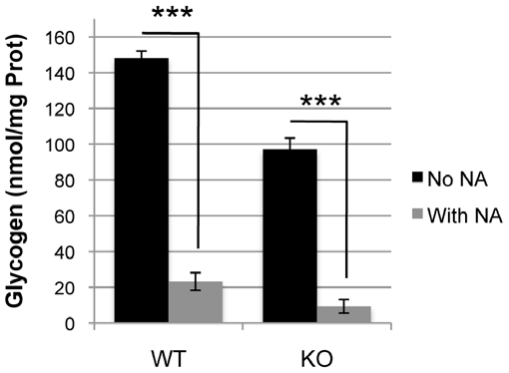
Effect NA treatment on glycogen mobilization in WT and GCLM-KO astrocytes. NA significantly induced a massive glycogen mobilization in both WT and KO astrocytes. n = 9 in WT and n = 6–8 in KO. *** p<0.001.

Under basal conditions, i.e. when no DAB and no stress are applied, the rate of glucose utilization was similar between WT and KO, while glycogen levels were lower in the KO astrocytes (Fig. 1, see above and Fig. 5). Following an oxidative challenge with 10 µM or 50 µM tBH, but in the absence of DAB, no significant change was noticeable in the uptake of glucose in both WT (Fig. 5A) and KO (Fig. 5B). In contrast to glucose utilization, a highly significant effect of treatment on glycogen levels could be observed. Indeed, in WT astrocytes, glycogen levels were significantly decreased after treatment with 10 µM tBH compared to control (−31.8%; p = 0.032). The decrease was even larger after 50 µM tBH (−88.6%; p<0.001). As for glucose utilization, glycogen levels were not affected by oxidative stress in KO astrocytes (Fig. 5D). To exclude the possibility that KO astrocytes are unable to mobilize glycogen, they were exposed to the well-established glycogenolytic agent noradrenaline [NA; 65] for 40 min. Results presented in Fig. 6 demonstrate that NA induced a massive glycogen mobilization in both WT and KO astrocytes (−84.3% and −90.4% respectively, p<0.001).

In order to determine if glucose uptake is affected when glycogen mobilization is impaired, cells were treated with DAB under basal and tBH-treated conditions. As expected, DAB prevented glycogen mobilization following tBH treatment in both WT (Fig. 5C) and KO astrocytes (Fig. 5D). In WT, prevention of glycogen mobilization is associated with higher rates of glucose utilization during oxidative stress (Fig. 5A). Indeed, the increase in glucose utilization was significant after treatments with 10 µM tBH (1.5-fold increase; p = 0.004) and 50 µM tBH (1.6-fold increase; p = 0.006) in the presence of DAB, compared to oxidative stress induced in the absence of DAB. In KO astrocytes, DAB induced a significant increase in glucose utilization (1.6-fold increase; p = 0.002) after treatment with 50 µM tBH although no increase was seen in response to 10 µM tBH.

## Discussion

The aim of the present study was to determine if a deficit in GSH would have an impact on glucose metabolism in cultured astrocytes. Our results show that there is no difference in basal glucose utilization, lactate release, and TCA and PPP activities in GCLM-KO astrocytes compared to WT astrocytes. However, basal glycogen levels are lower in astrocytes from KO mice and the mobilization of glycogen following oxidative stress appears to be disturbed in these cells.

### Glycogen levels are lower in astrocytes from GCLM-KO mice

Basal glycogen levels are lower in KO astrocytes and blocking the breakdown of glycogen for 24 h with DAB revealed a higher turnover in KO than in WT cells. This lower glycogen level and higher glycogen turnover in KO astrocytes is accompanied by a decrease in the expression of the genes coding for key enzymes implicated in the synthesis and degradation of glycogen; namely, PTG, GS and GPhos. PTG is a protein that interacts with GS and GPhos, and sets glycogen metabolism to a synthetic mode [66], [67]. The decrease in the expression of the genes coding for proteins implicated in the synthesis and degradation of glycogen appears to be related to the low levels of glycogen observed in GCLM-KO mice. This observation is in line with results obtained from a study using PTG heterozygous mice and showing decreased glycogen stores in adipose tissue, liver, heart, and skeletal muscle in these mice compared to WT mice [68]. However, to our knowledge, this is the first demonstration that chronically low GSH levels and/or oxidative stress could affect PTG gene expression. Despite the clear involvement of PTG in glycogen metabolism, how its function is regulated is not known [69].

### In WT astrocytes, glycogen is mobilized following oxidative challenge

Phenotypes related to a defect in the genes coding for the enzymes required for GSH synthesis can be revealed under conditions of oxidative stress [64]. Cultured astrocytes were therefore submitted to a treatment with tBH, a ROS-generating agent. After 40 minutes of this treatment, GSH and GSSG levels were maintained at normal values in WT astrocytes, showing that the GSH used to detoxify the tBH had been regenerated through the redox cycle. During GSH peroxidase-catalyzed reduction of tBH, the ratio of GSH to GSSG is decreased and in order to re-establish a normal ratio, GSSG must be reduced by GSH reductase [56]. For this reaction to occur, production of the reduced equivalent NADPH through the PPP is necessary [14]. Glucose 6-phosphate, which is used as the primary substrate for PPP, can be obtained by the direct phosphorylation of glucose by hexokinases or by degradation of glycogen and the subsequent action of phosphoglucomutase. Rahman and collaborators [70] have demonstrated that in astrocytes, glycogen is mobilized following an oxidative insult. They proposed that NADPH regeneration via PPP could, at least in part, be met by glycogen mobilization. In line with these results, we observed that glucose utilization was not changed in WT astrocytes following tBH treatment, while there was a concentration-dependent increase in glycogen mobilization. Such a metabolic response appears important to detoxify oxidative compounds and to maintain GSH levels during a short stress period such as the one used in the present study. More massive and sustained oxidative stress may overcome such astrocytic defense mechanisms and thus impair cellular metabolism and viability [71].

### In GCLM-KO astrocytes, glycogen mobilization is unchanged following oxidative challenge

Unlike WT, KO astrocytes were unable to mobilize glycogen to detoxify ROS. This observation is puzzling, particularly in view of the fact that treatment with NA led to a near-complete depletion of glycogen in both WT and KO. Therefore, the absence of an effect of tBH on glycogen mobilization in KO astrocytes cannot be attributed to the inability of these cells to mobilize glycogen. In these cells, GSH levels were also significantly lower following tBH treatment compared to the non-stress condition, indicating that the GSH used to detoxify the ROS could not be regenerated. A possible explanation for this observation is that since no increase in the mobilization of glycogen occurred, it is likely that not enough *de novo* NADPH was produced via the PPP. Following treatment with 10 µM tBH, although GSH was oxidized in the tBH detoxification process, no change in the redox ratio of GSH (amount of GSSG in relation to GSH) was observed, indicating that GSSG was probably released in the extracellular compartment [72]. However, after treatment with 50 µM tBH, the ratio GSSG/GSH was not maintained any longer indicating that cells were under oxidative stress, although cell death was not significantly increased as reflected by the levels of LDH released in the medium. It has been shown that decreasing GSH levels in cortical cultures causes the suppression of oxidative stress-induced activation of the PPP [73]. This suggests, along with the observation that glycogen is normally mobilized following treatments with oxidative stressors [this study and 70], that a metabolic coupling between oxidative stress, activation of PPP and glycogen mobilization exists in astrocytes. This coupling may be lost or impaired in GCLM KO cells. However, the lower levels of basal glycogen in KO astrocytes and the fact that these cells do not mobilize glycogen following an oxidative stress indicate that they developed an alternative, and still not fully elucidated, defense strategy such as the release of GSSG into the extracellular space (see above), even if the implication of other defense strategies cannot be excluded.

### Differential effect of DAB in WT and GCLM-KO astrocytes

When DAB, an inhibitor of the breakdown of glycogen, was present, glucose utilization was increased in WT following the oxidative challenge with tBH. This observation indicates that in the absence of glycogen as a source of energy, WT astrocytes were able to compensate this deficiency by increasing their glucose utilization, demonstrating the high plasticity of this cell type with regard to energy metabolism. The situation in KO astrocytes is quite different. Forty minutes after the treatment with 10 µM tBH, the uptake of glucose in the presence of DAB was unchanged. In these cells, DAB led to an increase in the uptake of glucose only with 50 µM tBH, a concentration that induced oxidative stress but no glycogen mobilization. Thus, in KO astrocytes a stimulatory effect of DAB on glucose utilization is observed under conditions for which no significant increase in glycogen mobilization takes place when no DAB was applied. This is in is in contrast to what is observed in WT astrocytes and such an apparent paradox can be resolved when taking into account the different rates of glycogen turnover in KO and WT astrocytes. Indeed, in WT astrocytes, DAB produces its effect on glucose utilization by preventing glycogen mobilization, whereas its effect on KO cells would rather be linked to blockade of glycogen turnover. The metabolic mechanisms by which glycogen turnover may become limiting in KO astrocytes only following a major oxidative stress (50 µM versus 10 µM tBH), and how an increase in glucose utilization could compensate for this, are not known. However, they highlight specific cellular adaptations of KO astrocytes with regards to glycogen metabolism in general and to changes in glycogen turnover in particular.

### GSH deficit and neuroenergetics in schizophrenia

Results presented here demonstrate major differences in relation to oxidative stress defense mechanisms between KO and WT astrocytes. Knowing that the end result of glucose metabolism is accompanied by the production of ROS, one can postulate that cellular adaptation towards oxidative stress in KO cells may partly involve a constraint/limitation of glucose utilization and glycogen mobilization when an oxidative challenge is already monopolizing the GSH system. Antioxidant defense system impairment and redox dysregulation [For recent reviews], [ see 19], [74,75], in particular GSH deficit [17]–[19], have been well documented in schizophrenia. In the frontal cortex of GSH deficit models, morphological abnormalities, such as a decrease in dendritic spine density is observed possibly reflecting an inability of astrocytes to protect neurons from oxidative stress in this dopamine-rich region [17], [76]. The present study shows that both glucose uptake (under stress condition) and glycogen metabolism are modified in astrocytes from a chronically GSH-deficient mouse compared to WT mice. In line with these results, altered glucose utilization is observed in prefrontal and anterior cingulate cortices of schizophrenia patients [77]–[80], and this decrease as been associated with cognitive impairment [81].

### Conclusion

Our main observation is that glycogen status and utilization are clearly modified in astrocytes from GCLM-KO mice. WT astrocytes show an increase in their glycogen mobilization, or glucose utilization if glycogen is not available, following an oxidative stress. However, KO astrocytes do not increase their consumption of energy substrates unless an extreme oxidative challenge occurs. This observation is likely to reflect an adaptation of the GCLM-KO astrocytes directed at reducing the production of ROS through the metabolism of glucose. More studies will be necessary to find out which source of energy these cells turn to when needed and what are the effects of such important disturbances in the GCLM-KO astrocytes on their neighboring neurons.
